# A Combined LD_50_ for Agrochemicals and Pathogens in Bumblebees (*Bombus terrestris* [Hymenoptera: Apidae])

**DOI:** 10.1093/ee/nvab139

**Published:** 2022-01-11

**Authors:** Harry Siviter, Alexander J Matthews, Mark J F Brown

**Affiliations:** Department of Biological Sciences, Centre for Ecology, Evolution, and Behaviour, School of Life Sciences and the Environment, Royal Holloway University of London, Egham, Surrey, TW20 0EX, United Kingdom; Department of Integrative Biology, University of Texas at Austin , 2415 Speedway, Austin, TX 78712, USA; Department of Biological Sciences, Centre for Ecology, Evolution, and Behaviour, School of Life Sciences and the Environment, Royal Holloway University of London, Egham, Surrey, TW20 0EX, United Kingdom; Fargro Limited, Vinery Fields, Arundel, BN18 9PY, United Kingdom; Department of Biological Sciences, Centre for Ecology, Evolution, and Behaviour, School of Life Sciences and the Environment, Royal Holloway University of London, Egham, Surrey, TW20 0EX, United Kingdom

**Keywords:** neonicotinoid, thiamethoxam, *Crithidia bombi*, *Bombus*, toxicity test

## Abstract

Neonicotinoid insecticides are the most commonly used insecticide in the world and can have significant sub-lethal impacts on beneficial insects, including bumblebees, which are important pollinators of agricultural crops and wild-flowers. This has led to bans on neonicotinoid use in the EU and has resulted in repeated calls for the agrochemical regulatory process to be modified. For example, there is increasing concern about 1) the underrepresentation of wild bees, such as bumblebees, in the regulatory process, and 2) the failure to determine how agrochemicals, such as neonicotinoids, interact with other commonly occurring environmental stressors, such as parasites. Here, we modify an OECD approved lethal dose (LD_50_) experimental design and coexpose bumblebees (*Bombus terrestris*) to the neonicotinoid thiamethoxam and the highly prevalent trypanosome parasite *Crithidia bombi*, in a fully crossed design. We found no difference in the LD_50_ of thiamethoxam on bumblebees that had or had not been inoculated with the parasite (*Crithidia bombi*). Furthermore, thiamethoxam dosage did not appear to influence the parasite intensity of surviving bumblebees, and there was no effect of either parasite or insecticide on sucrose consumption. The methodology used demonstrates how existing ring-tested experimental designs can be effectively modified to include other environmental stressors such as parasites. Moving forward, the regulatory process should implement methodologies that assess the interactions between agrochemicals and parasites on non-*Apis* bees and, in cases when this is not practical, should implement post-regulatory monitoring to better understand the real-world consequences of agrochemical use.

Neonicotinoids are systemic insecticides that are effective at controlling a broad range of pest species such as aphids, whiteflies, and pollen beetles (Simon-Delso et al. 2015). As neurotoxins they target the insect nervous system, acting as agonists of nicotinic acetylcholine receptors (nAChRs) (Moffat et al. 2016). Neonicotinoids can be used as a seed treatment or foliar spray, but are highly persistent in the environment, and may persist in soil for over a year (Goulson 2013, Bass et al. 2015, Bonmatin et al. 2015). Neonicotinoids can therefore contaminate the nectar and pollen of treated crops as well as neighbouring wildflowers, leading to exposure for bees and other flower visiting insects (Stewart et al. 2014, Botías et al. 2016). An analysis of global honey samples revealed that 75% of honey contained at least one neonicotinoid insecticide, with 45% containing two, confirming that bees are routinely exposed to neonicotinoids on a global scale (Mitchell et al. 2017). Such exposure can have significant negative effects on bee colony health, behaviour, and physiology (reviewed by Godfray et al. 2014, Goulson et al. 2015, Pisa et al. 2017, Main et al. 2018, Siviter et al. 2018b) which has led to bans and restrictions on their use globally, most notably in the European Union, where 3 commonly used neonicotinoids (imidacloprid, thiamethoxam, and clothianidin) are now banned. However, neonicotinoid use remains common globally, particularly in the United States and China (Simon-Delso et al. 2015).

Bumblebees are important pollinators of agricultural crops and wildflowers (Willmer et al. 1994, Garibaldi et al. 2013). Bumblebee nests routinely contain a plethora of different parasites and pesticides, suggesting that simultaneous exposure to both parasites and agrochemicals is the norm, not the exception (Goulson et al. 2018, Nicholls et al. 2018). When bees are exposed to multiple stressors, the stressors can interact and become more detrimental than when exposed to a stressor in isolation (Doublet et al. 2015, Tosi and Nieh 2019, Linguadoca et al. 2021). For example, Di Prisco et al. (2013) found that honeybees (*A. mellifera*) exposed to the neonicotinoid clothianidin had a reduced immune defence, which promoted the replication of DWV. Furthermore, coexposure to neonicotinoids and parasites can also increase the likelihood of adult, or larval mortality (Fauser-Misslin et al. 2014, Doublet et al. 2015). Therefore, understanding how, and to what degree, insecticides and parasites interact when bees are simultaneously exposed to both is of utmost importance.

Thiamethoxam is one of the most commonly used neonicotinoids in the world, and is routinely found in the nectar and pollen collected by bumblebees (Botías et al. 2017, Nicholls et al. 2018). *Crithidia bombi* is a trypanosome parasite that is highly prevalent in bumblebee populations, with infection levels ranging from 0 to 80%, depending upon the population and time of year (Shykoff and Schmid-Hempel 1991, Gillespie 2010, Kissinger et al. 2011, Jones and Brown 2014). *C. bombi* exposure when combined with stressors like nutrient limitation or hibernation can significantly reduce bumblebee survival (Brown et al. 2000), colony founding, growth and reproductive output (Brown et al. 2003, Yourth et al. 2008), and can also impair foraging behaviour and learning (Gegear et al. 2005, 2006; Otterstatter et al. 2005) but see (Martin et al. 2018). Previous studies investigating the interactions between thiamethoxam and *C. bombi* have shown various interaction effects (Fauser-Misslin et al. 2014, Fauser et al. 2017, Baron et al. 2017) and simultaneous exposure to both stressors can lower bumblebee queen survival (Fauser-Misslin et al. 2014). This suggests that toxicity assessment of thiamethoxam conducted in the regulatory process could underestimate the potential real-world consequences of thiamethoxam exposure on bumblebees infected with common bumblebee parasites.

Agrochemical regulatory processes differ between nations and governing bodies. The European Union, which is considered to have the most rigorous regulatory process, has a tiered system that is heavily reliant on toxicity tests in the lower tiers to determine whether agrochemicals (pesticides, insecticides, fungicides, herbicides) are hazardous to animals (EFSA 2013, OECD 2017, Sanchez-Bayo and Tennekes 2017). When determining whether an agrochemical is ‘bee safe’ or not, toxicity tests, such as LD_50_ and LC_50_ tests will be conducted on honeybees (Tier 1) to determine the amount of active ingredient that is required to kill 50% of the population when bees are orally (LD_50_) or topically (LC_50_) exposed. Based on this information, further higher tier assessments will, or will not, be conducted (EFSA 2013, Sanchez-Bayo and Tennekes 2017). In its current form bumblebee LD_50_ experiments can be conducted in Tier 1 of the regulatory process, but this is not mandated, and the potential interactions between insecticides and other environmental stressors are not considered (EFSA 2013, Sanchez-Bayo and Tennekes 2017). Regulators and policy makers therefore require methodologies that can be used within the current regulatory framework that 1) assess the impact of agrochemicals on non-*Apis*-bees and 2) test how agrochemicals interact with other environmental factors (EFSA 2013, Vanbergen & Insect Pollinators Initiative 2013, Franklin and Raine 2019; Siviter et al. 2021a, c).

Here we ask if simultaneous exposure to both thiamethoxam and *C. bombi* changes the LD_50_ values of thiamethoxam in bumblebees (*Bombus terrestris*). The acute, oral LD_50_ for bumblebees (*B. terrestris*) and thiamethoxam is known to be 5 ng of active ingredient per bee (EFSA 2015) and so if thiamethoxam and *C. bombi* significantly interact we would predict that this value would either increase or decrease. Our methodology was based on OECD guidelines (OECD 2017) but was modified to incorporate *C. bombi* inoculation. We hypothesised that when used in combination, thiamethoxam and *C. bombi* would lower the LD_50_ value of bumblebees (*B. terrestris*).

## Methods

Six bumblebee colonies (*Bombus terrestris audax*) were ordered from Agralan (United Kingdom) and transferred into plastic colony boxes (28 × 22 × 12 cm) and maintained in a laboratory (25°C & 42% humidity), with ad libitum access to sucrose solution (50°Brix) and pollen (Agralan). The faeces of 15 workers from each colony were examined using a phase contrast microscope for common bumblebee parasites (*Apicystis bombi*, *Crithidia spp.* & *Nosema spp.* 400× magnification) (Rutrecht and Brown 2009). All colonies were unparasitized.

### Parasite Inoculation

The aim of this experiment was to determine if inoculation with the parasite *C. bombi* changed the LD_50_ of thiamethoxam on bumblebees. To achieve this, we had a total of 21 treatment groups (2 control groups, 1 *C. bombi* group, 9 thiamethoxam groups and 9 groups exposed to both thiamethoxam & *C. bombi*; see Supp Table S1 [online ony]). We had 40 bumblebees in each treatment group and all bees were individually housed in Nicot cages (see below for details).

To create a *C. bombi* inoculum the faeces of 30 workers were taken from a commercial colony infected with multiple strains of *C. bombi*. These strains were originally isolated from bumblebee queens caught at Windsor Great Park (United Kingdom) and then propagated through commercial colonies in the laboratory. Faeces of infected workers from these colonies were placed in an Eppendorf tube containing 0.9% Ringer solution and centrifuged at 0.8 g for 2 min. The supernatant was removed, and clean Ringer solution added, a process that was repeated 7 times (8 times in total) to purify and concentrate the preinoculum (following a modified triangulation protocol based on [Cole 1970]). Cell counts were carried out using a Neubauer improved haemocytometer to determine the concentration of *C. bombi* cells. *The C. bombi* preinoculum was then combined with sucrose (50°Brix) to create an inoculum of 1,000 cells/ul.

Individual bumblebees from all treatment groups (see Supp Table S1 [online only] for list of treatment groups) were taken from queen-right colonies, and individually housed in Nicot cages (148 × 130 × 11 mm) with ad libitum access to 50°Brix sucrose through a 1 ml syringe.

Prior to inoculation, workers from all the treatment groups underwent a starvation period of 3 h (Logan et al. 2005) after which all bees were removed from their Nicot cages and placed in an individual vial (9 × 2.5 cm). The inoculum was presented to each individual to drink with a 10 µl droplet of 50°Brix sucrose solution containing approximately 10,000 *C. bombi*. A dose of 10,000 cells has been determined to produce a reliable and high rate of infection (Ruiz-González and Brown 2006). A period of 15 min was allowed for the individual to consume the inoculum. Workers from control and thiamethoxam only treatment groups underwent the same procedure but were presented with a 10 µl droplet of sucrose solution (50°Brix). All workers were then placed back into their allotted Nicot cages and the sucrose syringe was returned. To enable the parasite to establish itself within the host the bees (both parasitized and unparasitized) were then left for 7 d  (Schmid-Hempel and Schmid-Hempel 1993, Logan et al. 2005). 76 bumblebees died during this time period, but there was no difference in mortality between inoculated and uninoculated bees (see Supp Table S1 [online only]).

### Thiamethoxam Exposure

Thiamethoxam PESTANAL analytical standard (100 µg) was purchased from Sigma-Aldrich and combined with 100 ml of acetone solution to produce the stock solution, which was subsequently combined with sucrose (50°Brix) to create the required dosages. The acute oral LD50 for thiamethoxam in *B. terrestris* has previously been determined to be 5 ng of active substance/bee (EFSA 2015) and we based our dosages on this (see Supp Table S1 [online only]).

Prior to being fed the relative thiamethoxam dose the sucrose syringes were removed from the Nicot cages and the bees starved for 3 h. Following this, the syringes were replaced with new ones with a 40 µl sucrose solution (50°Brix) containing the relevant thiamethoxam dosage. Bees were left for 4 h, after which the syringes were replaced with weighted syringes containing clean sucrose. Bees that had not consumed the entire dosages were removed from the experiment (*n* = 8, see Supp Table S1 [online only]).

Bees were left for 96 h and mortality was recorded at 4, 6, 8, 24, 48, 72, and 96 h, after being fed the thiamethoxam inoculation (OECD 2017). All bees that died during the experiment were frozen at −80°C.

### Parasite Analysis

All bees were screened for *C. bombi* infection. Individual bees were dissected, and the hindgut was removed and placed into a 1.5 ml Eppendorf tube. 100 µl of 0.9% Ringer's solution was added and the hindgut was pulverised within the Ringer solution. The contents were then vortexed for 2 s. Uninoculated bees were checked for infection by placing 14 µl from each sample onto a microscope slide and analysing it for *C. bombi* cells under phase contrast at 400× magnification. No uninoculated bees were found to be infected.

For inoculated bees, we used a Neubauer improved haemocytometer to measure *C. bombi* intensity, and to count the number of *C. bombi* cells per µl. Inoculated bees that had no sign of an infection were removed from the analyses (*n* = 4).

Thorax width, as a proxy for body size, was measured using a Mitutuyo digital calliper, with all individuals measured three times to produce a mean measure of size.

### Statistical Analysis

We used an information theoretic model selection approach for each test (except for determining the LD_50_ values [see below]). The initial model set contained all measured factors and was compared to all subsets of the full model, and a null model containing just the intercept and random factors. Models were selected based on Akaike weights derived from AICc values, and were included when they could not be rejected with a 95% certainty (this included cases in which the null model was accepted within the confidence set). When more than one model was present within the confidence set, model averaging was used (Burnham and Anderson 2002).

Following Ritz et al. (2015) we used a fitted dose-response model (*drc*) based on a log-logistic regression analysis to determine the LD_50_ values for bumblebees that were and were not inoculated with *C. bombi.* A mixed effect Cox model and a linear mixed effect model were used to determine if *C. bombi* influenced bumblebee mortality and sucrose consumption respectively. *C. bombi*, thiamethoxam dosage, and their interaction were included as fixed factors and bee size was included as a covariate. Colony of origin was included as a random factor. Parasite count was logged (log10) to improve model fit and analysed using a linear mixed effect model with thiamethoxam dosages, with bee size included as a covariant, and colony of origin included as a random factor.

We used the packages *drc*, *MuMin*, *lme4* & *coxme* (Bates et al. 2015, Ritz et al. 2015, Barton 2016, Therneau 2018).

## Results

**Fig. 1.**
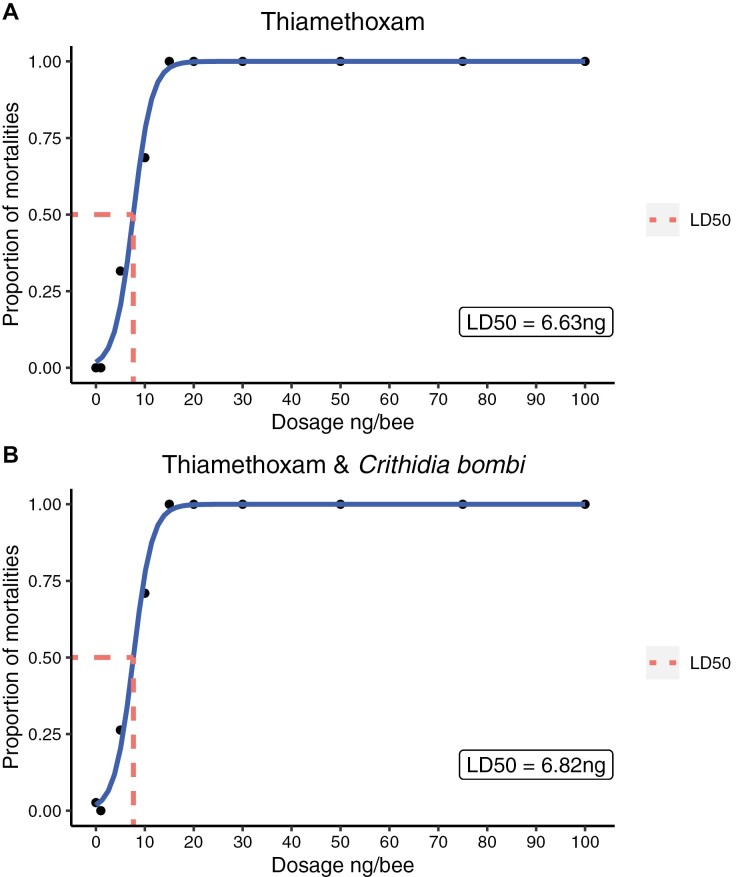
Dose-dependent plots demonstrating the LD_50_ values for bees exposed to thiamethoxam in isolation (A) and bees inoculated with *C. bombi* and exposed to varying dosage of thiamethoxam (B). We found no difference in the LD_50_ between parasitized and unparasitized bees.

**Fig. 2.**
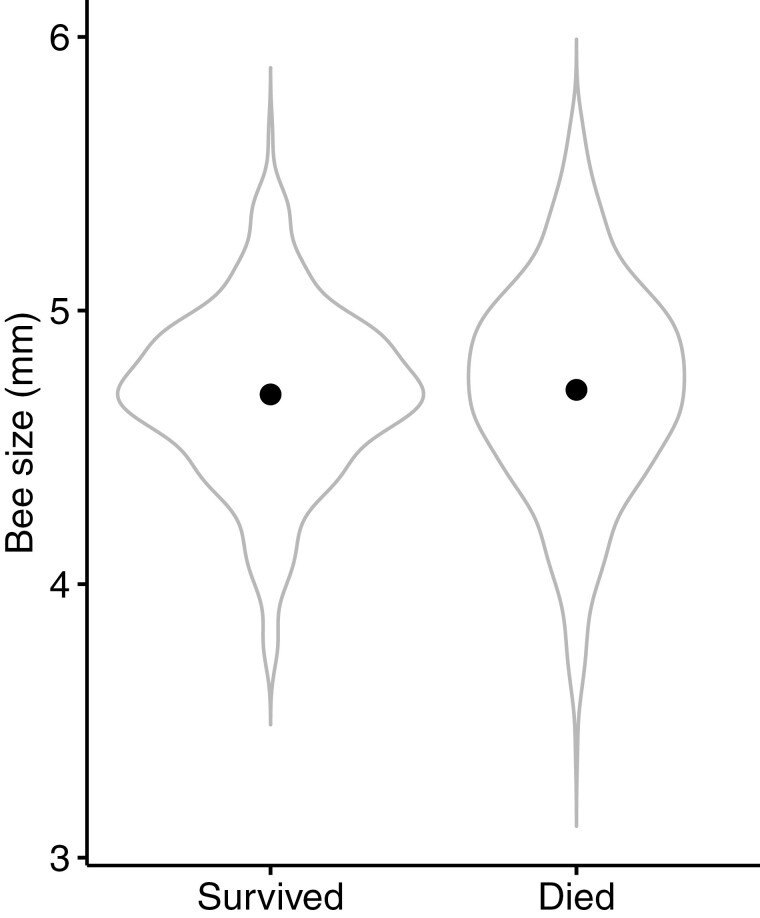
Violin plots depicting the average size (mm) of bumblebees that either survived or died during the experiment (96 h). Mortality risk was higher for both smaller and larger bees.

We found that the LD_50_ value for thiamethoxam was 6.63 ng when used in isolation compared with 6.82 ng per bumblebee when used in combination with the parasite *C. bombi*, suggesting no observed differences in mortality between infected and uninfected bumblebees (Fig. 1A and B, Coxme, *C. bombi*, Parameter Estimate (ES) = 0.10, Confidence Interval (CI) = −0.11 to 0.33). Bumblebee size had an effect on mortality, but the effect was not linear, with mortality risk increasing for both smaller and larger bees (Fig. 2, Coxme, size, PE = −0.35, CI = −0.60 to −0.11).

**Fig. 3.**
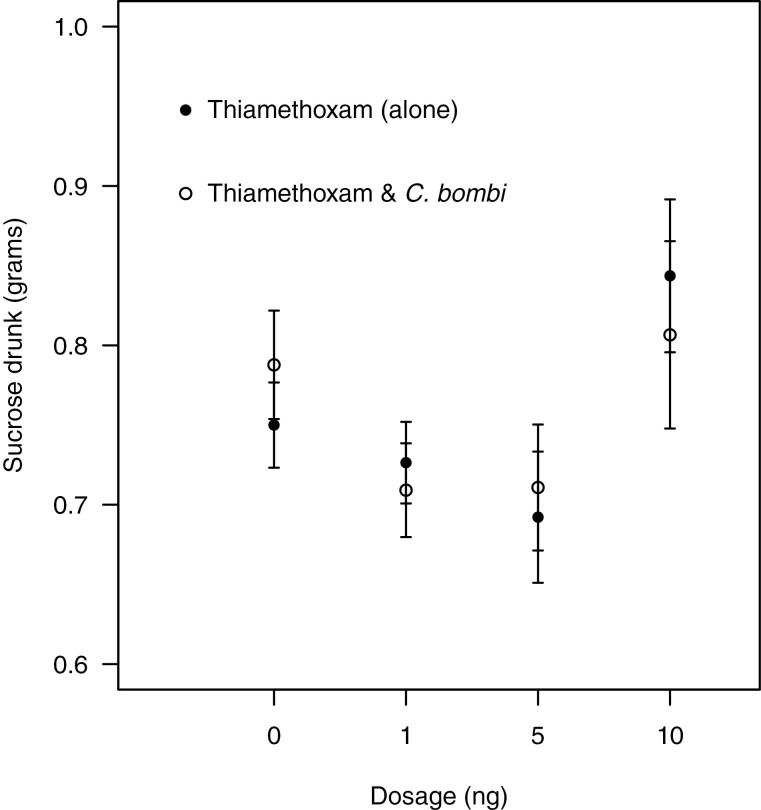
The mean amount (grams) of sucrose drunk (±SE) over 96 h from parasitized and unparasitized bumblebees (*C. bombi*) acutely exposed to varying dosages of thiamethoxam. Subjects that did not survive the experiments were excluded from this analysis.

**Fig. 4.**
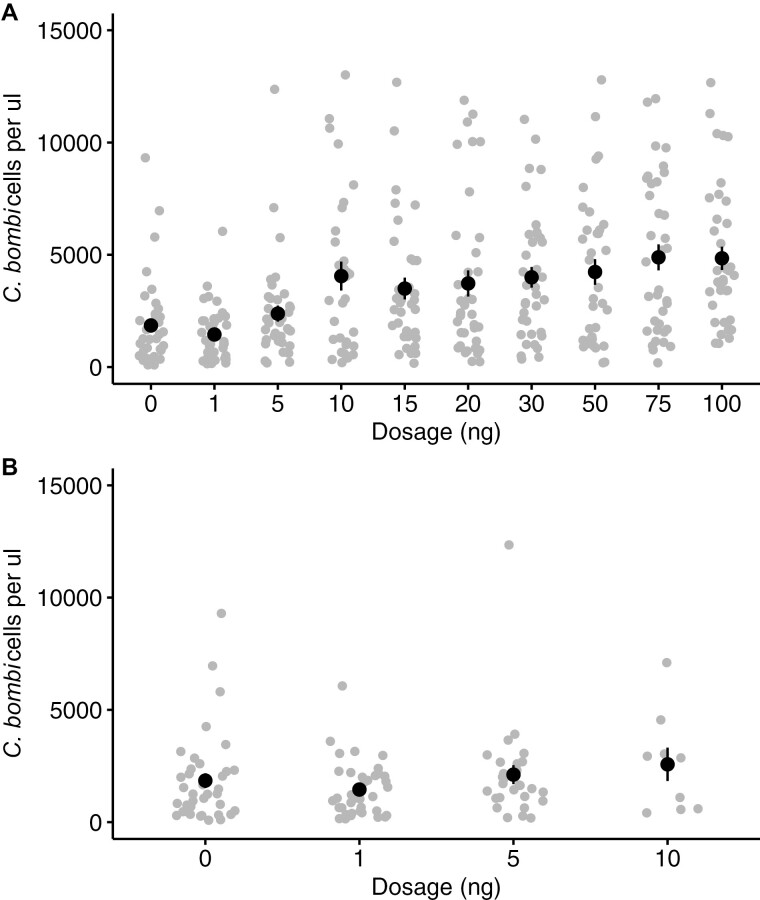
The mean (±SE) number of *C. bombi* cells per µl found in the hindgut of all bumblebee workers from the experiment (A) and only bumblebees that survived until the end of the experiment (B).

We found no effect of thiamethoxam or *C. bombi* inoculation on sucrose consumption (Fig. 3, Supp Table S2 and S3 [online only]). Interestingly, as thiamethoxam dose increased, this resulted in bumblebees having a higher intensity of *C. bombi* infection (Fig. 4A, lmer, dosage, PE = 0.0047, CI = 0.003–0.006). However, when subjects that died during the experiment were excluded from the analysis there was no effect of thiamethoxam dose on parasite intensity (Fig. 4B, lmer, dosage, PE = 0.001, CI = −0.01 to 0.01), suggesting no effect of thiamethoxam on *C. bombi* intensity at sub-lethal levels.

## Discussion

Previous studies with bumblebees have shown that the LD_50_ of thiamethoxam is 5 ng of active ingredient per bumblebee (EFSA 2015), and our results were similar (6.63 ng when exposed to thiamethoxam in isolation and 6.82 ng for bumblebees exposed to both thiamethoxam and *C. bombi*). This suggests that contrary to our original hypothesis, the parasite *C. bombi* had no impact on the LD_50_ of thiamethoxam on bumblebees (*B. terrestris*). This is surprising, as the effects of this parasite on bumblebees are context-dependent, and emerge most obviously when bees are exposed to other stressors (Brown et al. 2000, 2003; Yourth et al. 2008). Interestingly, and in contrast to previously observed results (Kessler et al. 2015, Arce et al. 2018) (but see [Muth et al. 2020]), we found no effect of thiamethoxam exposure on sucrose consumption. Finally, thiamethoxam exposure was seen to increase *C. bombi* intensity, but only at lethal dosages as there was no effect at sub-lethal levels. Our results demonstrate that methodologies currently used within the regulatory process can be modified to consider the interaction effects between multiple environmental stressors on wild bees.

We found no evidence of interaction effects between thiamethoxam and *C. bombi* on bumblebee mortality. This contrasts with previous studies that have shown that simultaneous exposure to both thiamethoxam and *C. bombi* can reduce bumblebee survival (Fauser-Misslin et al. 2014). However, Fauser-Misslin et al. (2014) assessed the impact of chronic, sub-lethal thiamethoxam concentrations over 9 wk on queen bumblebee survival, while here we used acute dosages, in a toxicity test with workers. Toxicity tests, such as LD_50_ experiments, are important in determining the lethal consequences of agrochemical use, but are not designed to detect more subtle, sub-lethal impacts of agrochemical exposure (Gill et al. 2012, Siviter et al. 2020b, Siviter et al. 2021b). While our modified LD_50_ protocol can be used to assess how parasites and agrochemicals interact at higher dosages, a failure to conduct sub-lethal assessments of chronic exposure in bumblebees alongside toxicity tests will clearly result in a failure to detect sub-lethal, but significant, interactions between agrochemicals and parasites (Fauser-Misslin et al. 2014, Siviter et al. 2021a). While our methodology could be used within the regulatory process, future research should be focused on developing methodologies that assess the potential sub-lethal interactions between agrochemicals and parasites on bees.

We found that *C. bombi* intensity was significantly higher in bumblebees that had been fed high dosages of thiamethoxam and that had subsequently died. *C. bombi* intensity typically increases for up to 7 d after inoculation and plateaus between 7- and 10-days post inoculation (Logan et al. 2005). We exposed bumblebees to thiamethoxam 7 d post inoculation and found that bees that died (on day 7) had a higher intensity of *C. bombi* than bees that survived (Fig. 4). One explanation for this is that acute exposure to thiamethoxam exerts long-term inhibition on the growth of *C. bombi*, and that this could only occur in bees that survived exposure. Alternatively, higher *C. bombi* counts in bumblebees exposed to lethal acute doses could be due to rapidly enhanced production or release of the parasite from the gut lining (Koch et al. 2019). Future experiments are needed to determine the mechanism behind this interaction. However, as we found no effect of sub-lethal thiamethoxam dosages on *C. bombi* intensity, this suggests that at field-realistic levels, thiamethoxam is unlikely to impact *C. bombi* intensity.

Neonicotinoids are the most commonly used insecticides in the world and understanding the interaction between them and bumblebee pathogens is therefore vitally important. However, as the number of insect pests that are resistant to neonicotinoids increase, and bans/restrictions on their use increase globally, novel insecticides such as sulfoxaflor or flupyradifurone could replace them over large geographical areas (Brown et al. 2016, Siviter and Muth 2020). Sulfoxaflor exposure can have significant sub-lethal impacts on bumblebee (*B. terrestris*) reproduction (Siviter et al. 2018a, Siviter et al. 2020 a,b; Linguadoca et al. 2021) (but see [Siviter et al. 2019]) and flupyradifurone exposure can impair honeybee larval development (Tan et al. 2017, Al Naggar and Baer 2019), and adult behaviour (Tosi and Nieh 2019, Tong et al. 2019, Hesselbach et al. 2020) (recently reviewed in [Siviter and Muth 2020]). Novel insecticides could also interact with bee pathogens, for example, bumblebee larvae fed sulfoxaflor in isolation showed no evidence of an increase in larval mortality, but when coexposed to sulfoxaflor, and the common bumblebee parasite *Nosema bombi*, there was a significant increase in larval mortality (Siviter et al. 2020a). Similarly, honeybees (*A. mellifera*) fed flupyradifurone and inoculated with *N. ceranae* had lower survival than unexposed bees, and those exposed to each stressor in isolation (Al Naggar and Baer 2019). While we found no interaction between *C. bombi* and the neonicotinoid thiamethoxam on bee mortality, future research should focus on understanding how novel insecticides, such as sulfoxaflor and flupyradifurone, interact with common bee parasites (Siviter and Muth 2020).

Global bee declines are thought to be driven by multiple anthropogenic stressors, including agrochemicals and parasites (Vanbergen & Insect Pollinators Initiative 2013, Goulson et al. 2015, Siviter et al. 2021a) which suggests that the agrochemical regulatory process should consider how insecticides interact with commonly occurring bee parasites (Siviter and Muth 2020). Here we show how toxicity tests, such as LD_50_ experiments, can be modified to consider the interactions between agrochemicals and parasites. This methodology could easily be modified to test other parasites depending on the life history of the parasites. However, the sheer number of bee parasites (known and unknown), and the range of different agrochemicals used in intensive agriculture means that testing every potential interaction between parasites and agrochemicals is impractical, and in some cases, when we do not have an understanding of the parasite life-history, impossible. In these cases, post-authorisation monitoring observations, which are currently nonexistent (Milner and Boyd 2017), should be carried out that monitor interactions between pesticides and pathogens. More broadly a move towards a more holistic approach to environmental risk assessment, that considers the interactions between multiple stressors, and models their impact on wild bees, is required to better safe-guard bees, and other pollinators, from the potential harm of agrochemicals (Siviter and Muth 2020, Topping et al. 2021).

## Supplementary Material

nvab139_suppl_Supplementary_Material

## Data Availability

Raw data available here https://osf.io/vautc/.
