# Supplementary material for: A Combined LD50 for Agrochemicals and Pathogens in Bumblebees (Bombus terrestris [Hymenoptera: Apidae])
Source: Environ Entomol. 2022 Jan 11;51(2):378–84. doi: 10.1093/ee/nvab139 (PMC9032631; doi:10.1093/ee/nvab139)

**Supplementary material: A combined LD_50_ for agrochemicals and pathogens in bumblebees (*Bombus terrestris*).**

Harry Siviter^1,2, †, *^, Alexander J Matthews^1,3†^, & Mark J F Brown^1^

^1^ Department of Biological Sciences, Royal Holloway University of London, Egham, Surrey, TW20 0EX, UK

^2^ Department of Integrative Biology, University of Texas at Austin, 2415 Speedway, Austin, TX 78712, USA

^3^ Fargro Limited, Vinery Fields, Arundel, BN18 9PY, United Kingdom

†These authors contributed equally

*corresponding authors ([Harry.Siviter.2016@live.rhul.ac.uk](mailto:Harry.Siviter.2016@live.rhul.ac.uk))

**Table S1: The 21 different treatment groups used in the experiment and sample sizes.**

| **Dose (ng/bee)** | ***C. bomi*** | **Number of bees at the start of the experiment** | **Bees that died before experiment** | **Bees that did not feed** | **Final sample size (n)** | **Mortalities** |
| --- | --- | --- | --- | --- | --- | --- |
| 0 (Control) | N | 40 | 5 | 0 | 35 | 0 |
| 0 (Negative control) | N | 40 | 5 | 0 | 35 | 0 |
| 0 (*C. bombi*) | Y | 40 | 2 | 0 | 38 | 1 |
| 1 | N | 40 | 3 | 0 | 37 | 0 |
| 1 | Y | 40 | 3 | 0 | 37 | 0 |
| 5 | N | 40 | 2 | 0 | 38 | 12 |
| 5 | Y | 40 | 2 | 0 | 38 | 10 |
| 10 | N | 40 | 5 | 0 | 35 | 24 |
| 10 | Y | 40 | 9 | 0 | 31 | 22 |
| 15 | N | 40 | 0 | 2 | 38 | 38 |
| 15 | Y | 40 | 1 | 4 | 35 | 35 |
| 20 | N | 40 | 3 | 1 | 36 | 36 |
| 20 | Y | 40 | 2 | 1 | 37 | 37 |
| 30 | N | 40 | 4 | 0 | 36 | 36 |
| 30 | Y | 40 | 3 | 0 | 37 | 37 |
| 50 | N | 40 | 5 | 0 | 35 | 35 |
| 50 | Y | 40 | 6 | 0 | 34 | 34 |
| 75 | N | 40 | 5 | 0 | 35 | 35 |
| 75 | Y | 40 | 4 | 0 | 36 | 36 |
| 100 | N | 40 | 4 | 0 | 36 | 36 |
| 100 | Y | 40 | 3 | 0 | 37 | 37 |


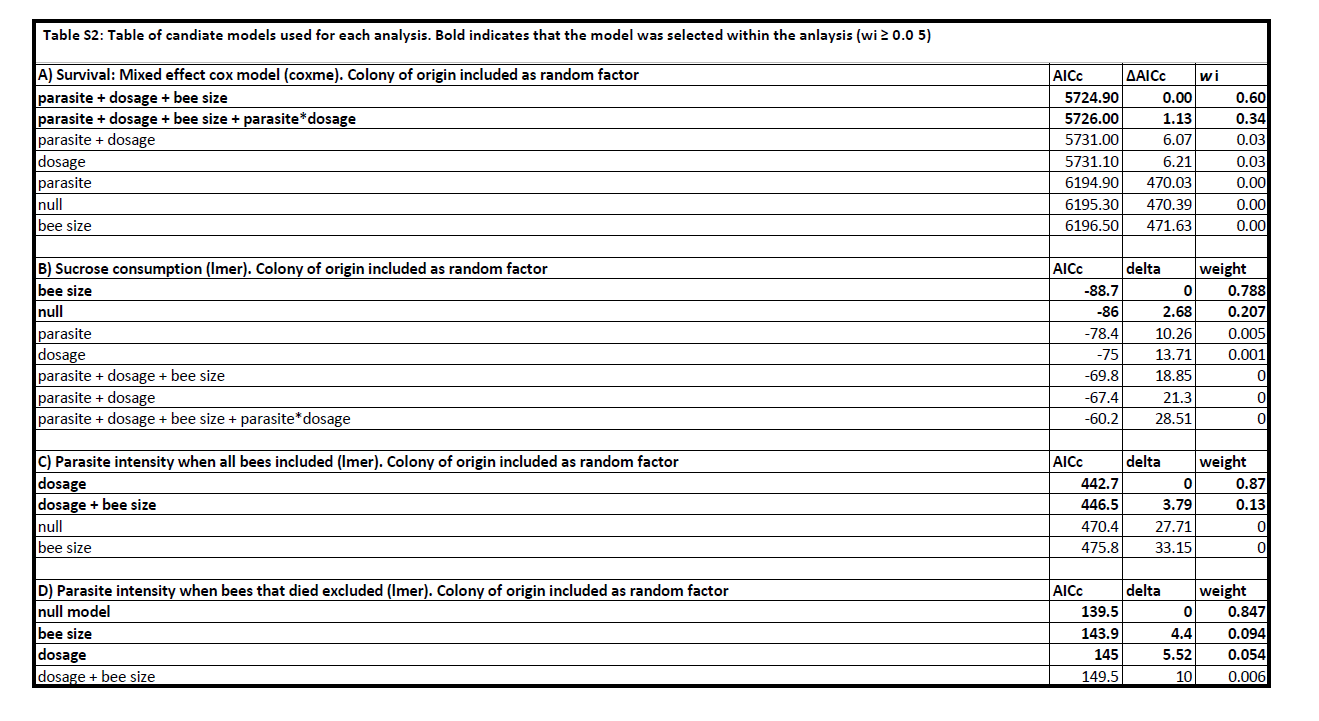


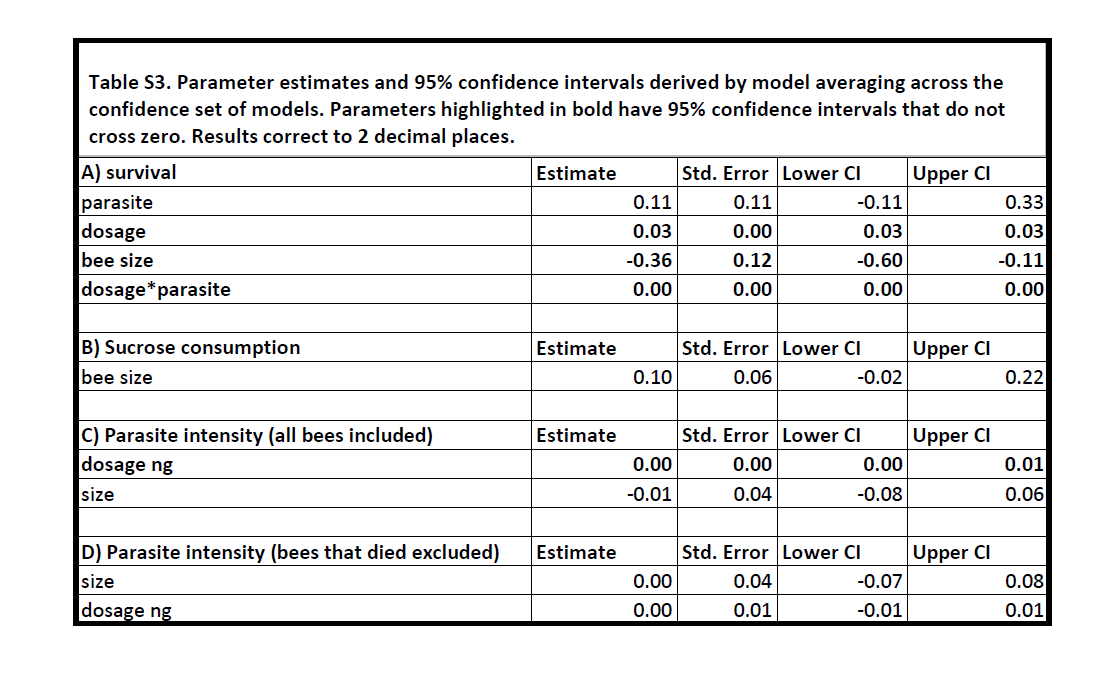

Supplement: nvab139_suppl_Supplementary_Material [file nvab139_suppl_supplementary_material.docx]
